# Differential Interaction of Peripheral Blood Lymphocyte Counts (ALC) With Different *in vivo* Depletion Strategies in Predicting Outcomes of Allogeneic Transplant: An International 2 Center Experience

**DOI:** 10.3389/fonc.2019.00623

**Published:** 2019-07-10

**Authors:** Vipul Sheth, Vanessa Kennedy, Hugues de Lavallade, Donal Mclornan, Victoria Potter, Brian G. Engelhardt, Bipin Savani, Wichai Chinratanalab, Stacey Goodman, John Greer, Adetola Kassim, Sally York, Michelle Kenyon, Shreyans Gandhi, Austin Kulasekararaj, Judith Marsh, Ghulam Mufti, Antonio Pagliuca, Madan Jagasia, Kavita Raj

**Affiliations:** ^1^Department of Haematology and Stem Cell Transplantation, Kings College Hospital, London, United Kingdom; ^2^Department of Haematology and Stem Cell Transplant, Stanford University, Stanford, CA, United States; ^3^Department of Haematology and Stem Cell Transplant, Vanderbilt University, Nashville, TN, United States

**Keywords:** antithymocyte globulin, alemtuzumab, allogeneic stem cell transplant, acute myeloid leukemia, absolute lymphocyte counts

## Abstract

Dosing regimens for antithymocyte globulin (ATG) and anti-CD52 antibody (alemtuzumab) for graft vs. host disease prophylaxis (GVHD) are empiric or weight-based, and do not account for individual patient factors. Recently, it has been shown that recipient peripheral blood absolute lymphocyte count (ALC) on the day of ATG administration interacts with the dose of ATG administered to predict transplantation outcome. Similarly, we wanted to analyze if the recipient ALC interacts with alemtuzumab dosing to predict outcomes. We retrospectively compared 364 patients, 124 patients receiving ATG (anti-thymocyte globulin) for GVHD prophylaxis, and undergoing unrelated first allogeneic transplant for myeloid and lymphoid malignancies (group 1) to 240 patients receiving alemtuzumab (group 2), in similar time period. There was no difference in survival or acute and chronic GVHD between 60 and 100 mg of alemtuzumab dosing. Unlike ATG (where the pre-transplant recipient ALC interacted with ATG dose on day of its administration (day 1) to predict OS and DFS (*p* = 0.05), within alemtuzumab group, the recipient ALC on second day of alemtuzumab administration (day 2) and its interaction with alemtuzumab dose strongly predicted OS, DFS and relapse (*p* = 0.05, HR-1.81, 1.1–3.3; *p* = 0.002, HR-2.41, CI, 1.3–4.2; and *p* = 0.003, HR-2.78, CI, 1.4–5.2), respectively. ALC (day 2) of 0.08 × 10^9^/lit or higher, had a specificity of 96% in predicting inferior DFS. Like ATG, there is definite but differential interaction between the recipient peripheral blood ALC and alemtuzumab dose to predict OS, DFS, and relapses.

## Key Points

- Current dosing of ATG and alemtuzumab for GVHD prophylaxis in hematological malignancies is empiric and does not take the drug target, the recipient lymphocyte, into consideration.
- The dose of alemtuzumab interacted with the recipient absolute lymphocyte counts (ALC) on day 2 of its administration to predict OS, DFS, and relapses, unlike ATG, wherein recipient ALC on the day of ATG administration (day 1) interacted with ATG dose to predict OS. Individualizing alemtuzumab dosing based on recipient ALC to improve outcomes without compromising immune reconstitution needs to be studied prospectively.
- Outcomes are comparable between two doses of alemtuzumab.

## Introduction

The therapeutic potential of allogeneic stem cell transplantation (allo HCT) remains limited by both acute and chronic graft-vs. host disease (GVHD) (1–3). There are multiple strategies aimed at preventing GVHD; however, there is currently no standard prophylaxis regimen (4).

Prophylactic *in vivo* T cell depletion with antithymocyte globulin (ATG), an antibody to the T cell receptor derived from either rabbit or horse sera, and alemtuzumab (monoclonal antibody against CD52), have both been effective strategies in reducing the incidence and severity of GVHD (5–8). The dosing regimens of both agents, however, are empiric and highly variable. An ideal dose should prevent severe GVHD, yet maintain graft- vs. -tumor effects, while simultaneously allowing for adequate immune reconstitution. Previous studies of high-dose ATG dosed at 10 mg/kg reduced the risk of GVHD but resulted in increased infectious complications and higher non-relapse mortality (9, 10). Subsequent, dose reductions to 6–8 mg/kg (11–13) decreased infectious complications, and a further reduction to 2–5 mg/kg was associated with a higher risk of GVHD (14, 15). Similarly, high-dose alemtuzumab dosing at 100 mg reduced the incidence of GVHD, but at the expense of delayed immune reconstitution (16), increased relapse (17), and increased opportunistic infections (18, 19). Reducing the dose to 30 mg was associated with improved lymphocyte recovery, while a further dose reduction to 20 mg was associated with an increased incidence of severe GVHD (20). Furthermore, the persistence of alemtuzumab in blood is variable and depends on the white blood cell count, and alemtuzumab clearance is based on CD52 expression and timing and mode of administration (21) instead of recipient body weight (22–24).

Recently, ATG was shown to interact with the recipient's peripheral blood absolute lymphocyte count (ALC) of the recipient on the day of administration to predict transplantation outcomes (25, 26). In contrast, in another recent study, it was shown that the recipient ALC on the day of ATG administration (day-2) was best predictive of ATG (AUC) exposure post-transplant and GVHD but not outcomes (27). We hypothesized similar effects would be observed for alemtuzumab. In this study, we retrospectively analyzed the interaction between the total amount of alemtuzumab administered and the recipient's ALC to predict outcomes. We also compared the outcomes of allo-SCT for patients receiving alemtuzumab 60 and 100 mg.

## Patients and Methods

We retrospectively analyzed 364 patients, of whom 124 (group 1, Vanderbilt University Medical Center, 2006–2013) received ATG and 240 (group 2, King's College hospital, UK; 2007–2014) received alemtuzumab. The cohort included patients with acute myeloid leukemia (AML), myelodysplastic syndrome (MDS), myeloproliferative disorder (MPN), lymphoma, lymphoproliferative disorders, and acute lymphoblastic leukemia (ALL). All patients received first allo-SCT with myeloablative or reduced intensity conditioning regimens from matched (10/10) or mismatched (9/10) unrelated donor grafts. The primary objective of this study was to analyze how the dose of alemtuzumab interacts with the recipient ALC to predict transplant outcomes, as compared to ATG. The secondary objectives were to compare overall survival (OS), and disease-free survival (DFS), the non-relapse mortality (NRM), relapse incidence and incidence of acute and chronic graft vs. host disease (aGVHD and cGVHD) between the two doses of alemtuzumab, and between ATG and alemtuzumab. This study was reviewed and approved by the Institutional Review Boards of Vanderbilt University Medical Center and Kings College hospital.

### GVHD Prophylaxis and Stem Cell Collection

The majority of patients (343/364, 94%) received peripheral blood stem cell (PBSC) grafts. In the alemtuzumab group, 80/240 (33%) patients received 60 mg of alemtuzumab (days -6 to -4) and 160/240 patients received 100 mg of alemtuzumab (days -8 to -4). In the ATG group, 67/124 (54%) patients received 5 mg/kg of ATG (days -2 and -1), 33/124 patients received 7.5 mg/kg (days -3 to -1), and 24/124 patients received 10 mg/kg (days -4 to -1). Alemtuzumab was given on days -3 to -1 for Bu/Cy and Cy/TBI regimens. Changes in dosing over time reflect systematic changes in institutional practice. In the alemtuzumab group, additional GVHD prophylaxis included a calcineurin inhibitor, and mycophenolate mofetil for ablative conditioning (Bu/Cy, Cy/TBI) and a calcineurin inhibitor alone for RIC and fludarabine-based myeloablative transplants (FB4, FB2, FM). Conditioning regimen consisted of fludarabine and high-dose busulfan (16 mg/kg orally or 12.8 mg/kg i.v.) (FB4) or cyclophosphamide (120 mg/kg) and total body irradiation (1200 cGy) (Cy/TBI) or busulfan (16 mg/kg, orally or 12.8 mg/kg i.v.) and cyclophosphamide (120 mg/kg) (Bu/Cy) for patients receiving ablative conditioning, and fludarabine and busulfan (6.4 mg/kg i.v.) (FB2), fludarabine, cyclophosphamide and rituximab (FCR), or fludarabine and melphalan (140 mg/m2) (FM) for patients receiving reduced intensity conditioning (RIC). The choice of conditioning regimen reflects institutional practice for varying disease histology. Pre-transplant disease risk (28), cytogenetic abnormality (29), and acute and chronic GVHD were defined as detailed in the Supplementary Materials (30–32).

### Statistical Methods

Categorical variables were summarized as frequency counts and compared using 2 × 2 tables and continuous variables were summarized as medians and compared using Mann Whitney *U*-test. OS was calculated from the time of transplant to the last follow-up date, DFS was calculated as the time from transplantation and the earliest occurrence of any event- relapse or death. OS, DFS, and GVHD were estimated by the Kaplan–Meier method and compared using the log-rank test.

Cumulative incidences of NRM and relapse between the ATG and alemtuzumab groups were compared using Fine and Gray model (33) and were considered as competing risks for each other (34). Following factors were considered for univariate analysis: disease type, disease risk, age, stem cell source, donor matching, type of conditioning, and *in vivo* depletion strategy. A multivariate model was constructed utilizing variables which were significant in univariate analyses (*p*-value < 0.2) or variables which were unequally distributed across two groups for each dependent outcome variable (OS, DFS, NRM, relapse or GVHD, **Tables 2**, **3**). Statistical analysis was conducted using SPSS 24.0 and R 3.4.3 software. All statistical tests were two-sided, and *p*-value 0.05 was used to indicate statistical significance. We performed sensitivity analyses of the ALC count from the day of starting administration of alemtuzumab through the day of transplant, and impact on transplant outcomes. Sensitivity and specificity of optimal ALC predicting DFS were obtained using receiver operating characteristic (ROC) curves.

## Results

### Demographic Profile

In the ATG group, 76 (61%) patients were older than 52 years compared to 151 (63%) patients in the alemtuzumab group. As compared to the ATG group, alemtuzumab group had significantly more number of patients receiving 9/10 mismatched donor grafts [6/124 (4%) vs. 56/240 (22%), *p* ≤ 0.001], patients with intermediate disease risk index [70/124 (57%) vs. 188/240 (78%), *p* < 0.001], higher exposure to myeloablative regimen [28/124 (22.6%) vs. 98/240 (40%), *p* < 0.001], more patients with myeloid disorder [85/124 (68%) vs. 203/240 (84%), *p* < 0.001], and more patients receiving peripheral blood stem cells [110/124 (89%) vs. 233/240 (97%), *p* = 0.005]. The median follow-up was 26 months (range, 1–98 months) in the ATG group and 33 months (range, 0.6–150 months) in the alemtuzumab group, respectively (Table 1).

**Table 1:** Demographic characteristic of our study cohort.

**Demographic profile**	**ATG (group 1) *N* = 124**	**Alemtuzumab (group 2) *N* = 240**	***p*-value**
**AGE (YEARS)**			
<52	48 (38%)	89 (37%)	0.95
>52	76 (62%)	151 (63%)	
**MATCHING**			
9/10	7 (5%)	56 (23%)	<0.001
10/10	117 (95%)	184 (77%)	
**DISEASE RISK INDEX**			
Intermediate	70 (56%)	188 (78%)	<0.001
High-risk	54 (44%)	52 (22%)	
**DISEASE TYPE**			
Myeloid	85 (68%)	203 (84%)	<0.001
Lymphoid	39 (32%)	37 (16%)	
**STEM CELL SOURCE**			
Peripheral blood	110 (88%)	233 (97%)	0.005
Bone marrow	14 (12%)	7 (3%)	
**CONDITIONING REGIMEN**			
Reduced intensity	96 (77%)	143 (60%)	<0.001
Myeloablative	28 (23%)	97 (40%)	

### Outcomes in Alemtuzumab Cohort

Within alemtuzumab group, the 2-year non-relapse mortality (NRM) was comparable between the two doses (18 vs. 24% for 60 and 100 mg, respectively, *p* = 0.37; **Figure 3A**). Bacterial infection (10 vs. 16%, *p* = 0.36) and viral reactivation (CMV/EBV and CMV disease) were similar for both 60 and 100 mg alemtuzumab (30 vs. 34%, *p* = 0.8, 29 vs. 30%, *p* = 0.9, 5 vs. 5%, *p* = 1.0, respectively). Acute GVHD (I-IV) and acute severe GVHD (grade III and IV) were comparable between both the two doses (Grade I-IV: 45 vs. 42%, *p* = 0.52; Grade III-IV: 12 vs. 10%, *p* = 0.8, for 60 and 100 mg, respectively). Similarly, 2 year extensive, severe, and all grade chronic GVHD were also similar between both the groups (Severe: 12 vs. 14%, *p* = 0.80; All-grade: 44 vs. 40%, *p* = 0.52, for 60 and 100 mg, respectively; **Figures 2A,B**). The median time to develop chronic severe GVHD was 11 months (range, 3–34 months).

**Figure 1:**
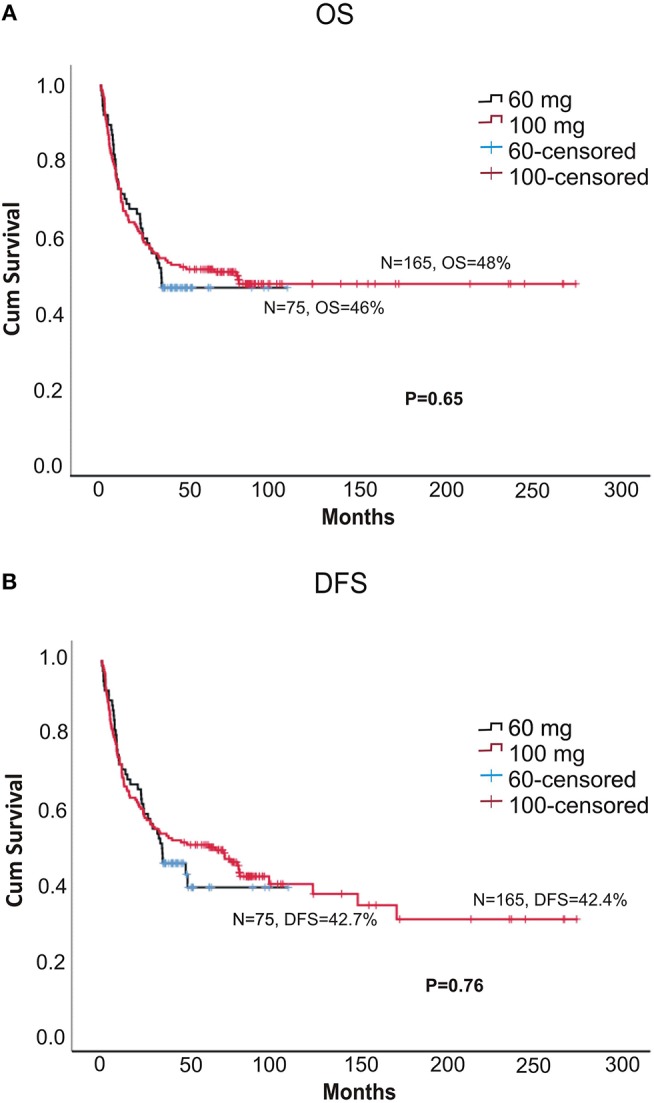
**(A,B)** The 5-year OS and DFS between different doses of alemtuzumab.

The 5-year OS (overall survival) and DFS (disease = -free survival) were comparable between the two doses (DFS: 42.7 vs. 42.4%, *p* = 0.76; OS: 46 vs. 48%, *p* = 0.66, for 60 and 100 mg, respectively; Figures 1A,B). The 2-year relapse rate was also similar between the two doses (31 vs. 38%, *p* = 0.22, 60 and 100 mg, respectively; Figure 3B). In multivariate analysis disease Risk Index, HLA matching, and ALC on day 2 significantly predicted relapse (Table 2).

**Figure 2:**
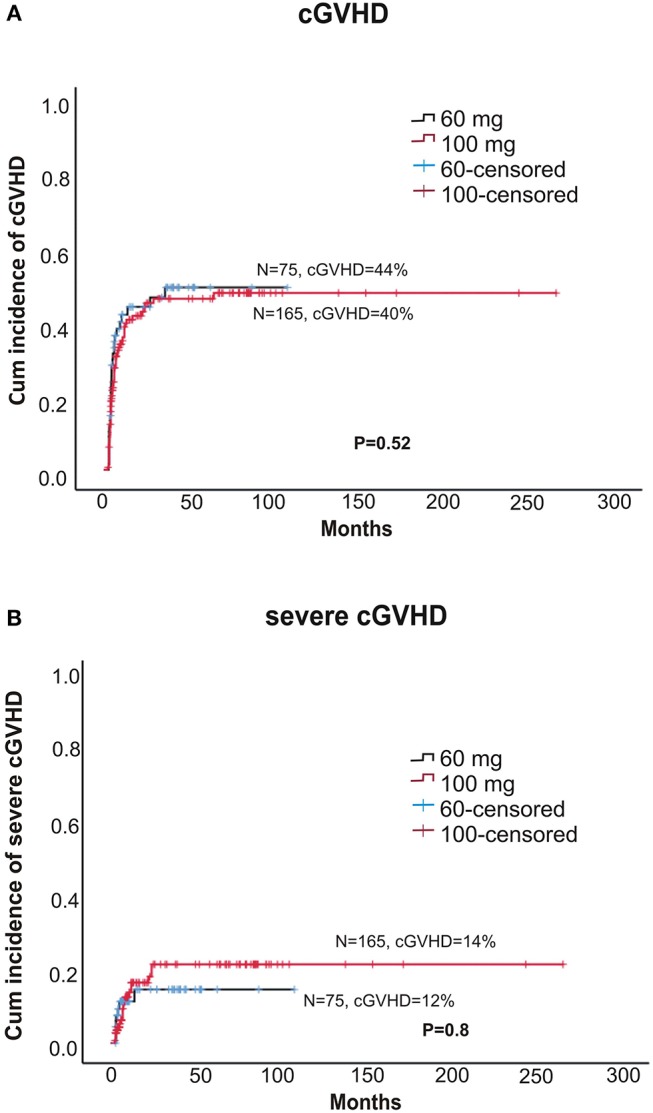
**(A)** Cumulative incidence of any grade chronic GVHD between two doses of alemtuzumab. **(B)** The cumulative incidence chronic severe GVHD between two doses of alemtuzumab.

**Figure 3:**
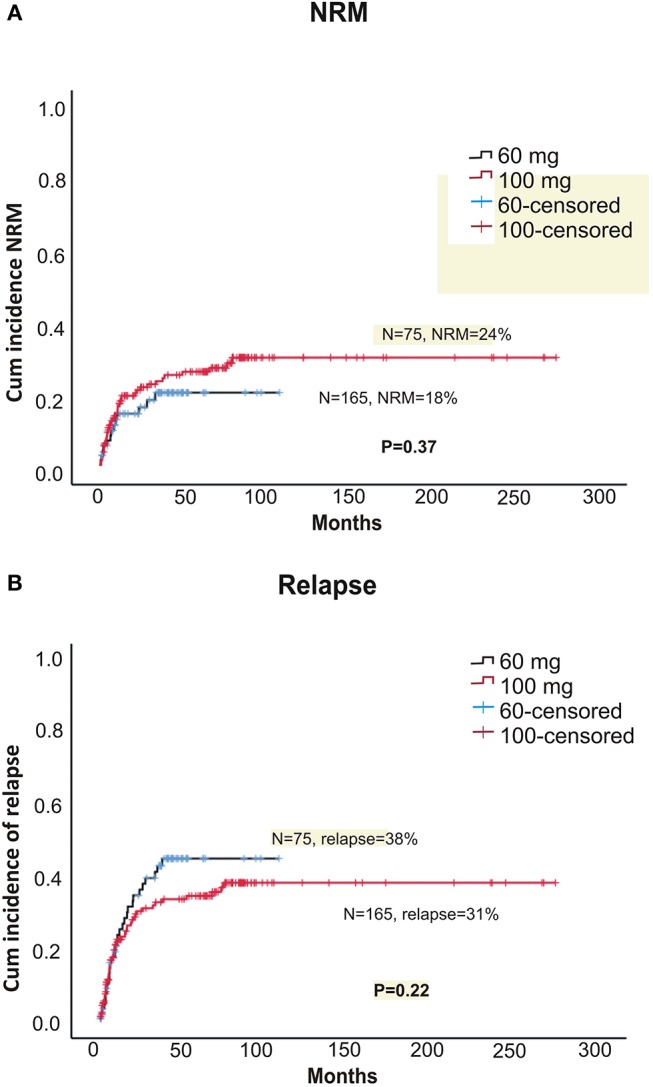
**(A)** Cumulative incidence of non-relapse treatment-related mortality (NRM) between two doses of alemtuzumab. **(B)** Cumulative incidence of relapse between two doses of alemtuzumab.

**Table 2:** Cox regression- Alemtuzumab group (group 2), variables affecting OS, DFS and relapse.

	**OS**			**DFS**			**Relapse**		
	**P**	**HR**	**CI**	**P**	**HR**	**CI**	**P**	**HR**	**CI**
Disease type (ref myeloid)	0.10	0.70	0.4–1.0	0.13	0.70	0.4–1.0	0.05	0.60	0.3–1.0
Age (ref >52 years)	0.0001	0.47	0.3–0.7	0.001	0.52	0.3–0.7	0.07	0.63	0.3–1.0
Matching (ref 10/10)	0.04	1.50	1.0–2.2	0.17	1.38	0.8–2.0	0.03	1.69	1.0–2.7
Conditioning (ref RIC)	0.62	1.09	0.7–1.6	0.21	1.24	0.8–1.7	0.79	0.94	0.5–1.5
ALC day 2	0.05	1.81	1.1–3.3	0.002	2.41	1.3–4.2	0.003	2.78	1.4–5.4
ALC day2/alemtuzumab dose	0.02			0.0001			0.001		
ALC day 1	0.2	0.85	0.6–1.1	0.21	0.84	0.6–1.1	0.11	0.73	0.4–1.0

*OS, overall survival; DFS, disease free survival; ALC, absolute lymphocyte counts of recipient; RIC, reduced intensity conditioning*.

### Multivariate Analysis and Interaction Between Total Dose Alemtuzumab and Recipient ALC

**Figure 4:**
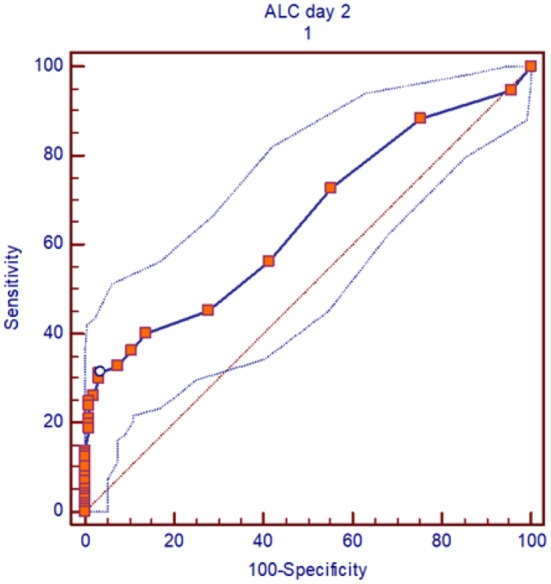
ROC curve, for pre-transplant recipient ALC of 0.08 × 10^3^/lit or more on day 2 of alemtuzumab administration, the outcomes (DFS) was particularly inferior. Area under curve (AUC) is 0.6 and sensitivity was 31% and specificity 96%.

In a multivariate analysis of the patients who received alemtuzumab, we found pre-transplant recipient ALC on second day of alemtuzumab administration (ALC day 2) as the strongest independent predictor of OS, DFS, and relapse (OS: *p* = 0.05, HR 1.81, 95%CI 1.1–3.3; DFS: *p* = 0.002, HR 2.41, 95%CI 1.3–4.2; relapse: *p* = 0.003, HR 2.78, 95%CI 1.4–5.2). The interaction between the total amount of alemtuzumab administered and ALC (ALC day2/alemtuzumab dose) was also an independent significant predictor of OS, DFS, and relapse (*p* = 0.02, *p* = 0.0001, and *p* = 0.001), respectively (Table 2). Based on the receiver operating characteristic, we determined ALC of 0.08 × 10^9^/L or higher on day 2 of alemtuzumab administration was a particularly strong predictor of poor DFS (sensitivity 31%, specificity 96%; Figure 4). There was no significant interaction between ALC and intensity as well as the type of conditioning regimen (data not shown).

### Differential Days of Interaction Between Total Dose ATG/Alemtuzumab and Recipient ALC

Within the ATG group, the pre-transplant recipient ALC (on the day of administration, day 1) predicted OS but not DFS (*p* = 0.05, HR-1.08, *p* = 0.2; Table 3). Also, the interaction between total amount of ATG administered and ALC was a significant predictor of OS (HR-0.9; *P* = 0.03). For an ALC at the 10th percentile (0.56 × 102/μL), higher total ATG administered was associated with a greater risk of death. However, unlike in ATG, we could not find any correlation between ALC day 1 and outcomes (Table 3). Furthermore, there was no interaction between ALC (day 1 or 2), alemtuzumab dose, and NRM, GVHD, or CMV reactivation and fungal infection (Supplementary Table 1).

**Table 3:** Cox regression for ALC and interaction with dose after multivariate analysis-ATG group and alemtuzumab group.

	**OS**			**DFS**			**Relapse**		
	**P**	**HR**	**CI**	**P**	**HR**	**CI**	**P**	**HR**	**CI**
ALC day 1 (ATG)	0.05	1.08	1–1.1	0.2	0.9	0.9–1.0	0.28	N.E.	
Interaction between ATG and ALC day 1	0.03	0.9	0.8–0.9	0.11	1.0	0.1–1.0	N.E.		
ALC day 2 alemtuzumab	0.05	1.81	1.1–3.3	0.002	2.41	1.3–4.2	0.003	2.78	1.4–5.4
Interaction between ALC and alemtuzumab dose	0.02			0.0001			0.001		
ALC day 1 (alemtuzumab)	0.2	0.85	0.6–1.1	0.21	0.84	0.6–1.1	0.11	0.73	0.4–1.0

*OS, overall survival; DFS, disease free survival; ALC, absolute lymphocyte count; N.E., P > 0.2 in univariate analysis*.

### Comparison of Outcomes Between ATG and Alemtuzumab

Overall, there is no significant difference in primary outcomes (OS, DFS, NRM, aGVHD, and cGVHD) between ATG and alemtuzumab group at all dose levels. However, the alemtuzumab group did have a lower incidence of early relapses (<2 years post-transplant) when compared to the ATG group (32 vs. 42%, *p* = 0.04, HR-1.47), probably due to implementation of timely pre-emptive DLI in alemtuzumab group (Supplementary Figures 1, 2, late merging of curves Figure 2B). However, in view of inter-center differences in strategy (pre-emptive DLI for alemtuzumab) and patient characteristics this could just be a preliminary evidence suggestive of equivalence between the two strategies, highlighting the importance of carrying out further prospective well-designed studies in future, in view of scarcity of data in this field.

## Discussion

Individualizing dose of ATG and alemtuzumab (*in vivo* T cell depletion) based on recipient characteristics is an unmet need for optimizing outcomes after allo- HCT. In view of difficulty in incorporating kinetics of ATG and alemtuzumab into regular clinical practice, there is a definite need for evaluating other accessible recipient-based factors such as ALC in predicting doses of *in vivo* depletion strategy. In the previous study, it was shown that the recipient peripheral blood ALC on the first day of ATG administration interacts with the weight-based dosing of ATG to influence OS (26). Our findings also suggest a definite interaction between blood recipient ALC and alemtuzumab dosing in predicting overall outcomes.

Overall, there is no significant difference in primary outcomes (OS, DFS, NRM, aGVHD, and cGVHD) between different dose levels of alemtuzumab. Furthermore, this study suggests that decreasing the dose of alemtuzumab does not significantly affect the risk of developing aGVHD or cGVHD or the rate of infectious complications. In our study, rates of acute and chronic GVHD are relatively higher, and cumulative incidence of early relapse were lower when compared to previous alemtuzumab studies (17, 20), among patients transplanted for hematological malignancy using reduced intensity conditioning regimen, and this may be due to the inclusion of patients receiving myeloablative regimens (42%), and implementation of pre-emptive DLI.

Interestingly, the interaction between the total amount of alemtuzumab and the pre-transplant recipient's ALC on second day of alemtuzumab administration (day 2) significantly predicted OS, DFS, and relapse. As per our study, an ALC count of 0.08 × 10^9^/lit or higher, as determined by the ROC curve, after first alemtuzumab administration (day 2) was associated with particularly inferior outcomes (OS, DFS). In ATG cohort, lower recipient ALC on day of administration of ATG (day 1) <0.05 × 10^9^/lit, meant lower target site for ATG, thus leading to persistence of ATG in recipient blood causing more immunosuppression, and poor outcomes (OS) though limited by lack of data on pharmacokinetic/dynamic (PK/PD) of ATG (25, 26). Similarly, we can hypothesize, that patients with high pre-transplant recipient ALC on day 2 of alemtuzumab would have had reduced binding of alemtuzumab to target recipient lymphocytes on day 1, thereby causing a higher amount of residual alemtuzumab to remain in the peripheral blood and subsequently lead to persistent immune suppression, relapse and poor outcomes (OS). Though conceptually very similar, we cannot possibly explain reduced and differential binding of alemtuzumab to target lymphocytes, and this would require further studies showing PK/PD properties of alemtuzumab, based on measuring its serial levels, along with differential CD52 expression in host lymphocytes (as has been shown previously in lymphoid malignancies (35).

Alternatively, there have been studies showing selection of CD52 negative population after alemtuzumab administration (36, 37) (unlike ATG being not selective for a particular target), in patients having lymphoid malignancies (37, 38) and auto-immune disorders (39). However, the functional characteristics of this population are unknown. In aplastic anemia, this population of persistent CD8 host lymphocytes been hypothesized in causing rejection of donor lymphocytes and eventually graft failure (40). Similarly, maybe the higher host ALC on day 2 might be CD52 negative selected population, leading to rejection of donor lymphocytes and eventually relapse, but this interesting finding needs to be studied prospectively in future.

In a kinetic model studied in a cohort of 137 patients undergoing cord blood transplant, area under the curve (AUC) of ATG after infusion of the CB predicted successful CD4+ IR and event free survival (EFS), with lower levels having better outcomes and graft vs. leukemia effect (41), as has also been shown in other studies receiving PB/BM as graft source (42, 43). In a recent cohort of 219 patients undergoing myeloablative transplant, it was shown that the lowest quintiles (higher recipient ALC on day of ATG administration leading to increased binding of ATG) of post-HCT AUC ATG were associated with inferior chronic graft-vs.-host disease and relapse-free survival (cGRFS). In this study, unlike previous data higher ALC predicted GVHD instead of outcomes, and the authors concluded that this discrepancy might be due to difference in doses, conditioning regimen and day of ATG administration, which contributes to ATG kinetics (27).

There is scarcity of literature regarding alemtuzumab. Our results are in accordance with the study (44), demonstrating interaction between pre-dose ALC and ALC area time with alemtuzumab levels on the day of transplant to predict survival and immunological recovery. In a previous study of pediatric patients by Marsh et al. (105 patients) (45), levels of alemtuzumab on day 0 of allogeneic graft was associated with the incidence of GVHD, mixed chimerism and lymphocyte recovery. An alemtuzumab level of <0.15 mmol/ml correlated with a higher recovery of lymphocyte subsets at day 30 and 100, and the authors proposed a model of adjusting alemtuzumab dosing to achieve optimal levels expected on the day of infusion.

Successful IR has been associated with survival outcomes, and graft vs. leukemia effects (GvL) (46–48). IR depends on the graft source (49, 50), as well as exposure to ATG (41) and alemtuzumab post-transplant (45). Reconstitution of naïve population (memory cells) is most important in protecting against infection (51) and providing beneficial GvL effect (52). Several studies showed that early post-transplantation ALC also correlates with immune reconstitution (IR) and significantly predicts transplantation outcomes, including GVHD (53–55). Though we do not have data on immunological reconstitution, like in ATG (26), surrogate markers for immune reconstitution, including NRM, infections, and cGVHD had no interaction of ALC with alemtuzumab dose (Supplementary Table 1). Lack of IR data remains a major drawback for this retrospective analysis.

The impact of other confounding interventions, such as type and intensity of conditioning regimen on ALC, remains a potential bias. We have tried to account for these potential effects by testing for the interactions between ALC and different intensities and types of conditioning regimen. Our study is also limited in that our analysis utilizes ALC, which includes both T cells and B cells, and also lacks data on PK/PD for alemtuzumab and ATG.

In summary, like ATG, there is definite but differential interaction between the recipient peripheral blood ALC and alemtuzumab dose to predict OS, DFS, and relapses. Pre-transplant recipient peripheral blood ALC on second day of alemtuzumab administration (day 2) was strongest predictor of outcomes, with higher ALC associated with inferior DFS, and one could possibly think of reducing doses of alemtuzumab (or maybe look into CD52 expression in persisting lymphocytes) over the forthcoming days, if the ALC on day 2 is higher than 0.08 × 10^9^/lit. Our approach has the potential to introduce new paradigm to study alemtuzumab dosing. Based on such an analysis, theoretically a norm gram of ALC and alemtuzumab dose could be developed prospectively, which could help in individualizing dose of alemtuzumab as per target recipient ALC, without compromising immune-reconstitution. Also, there is a need for further prospective PK/PD modeling studies for alemtuzumab/ATG in analyzing differential days of interaction with recipient ALC. Outcomes were comparable between 60 and 100 mg of alemtuzumab.

## Data Availability

The datasets generated for this study are available on request to the corresponding author.

## Ethics Statement

This study was carried out in accordance with the recommendations of Declaration of Helsinki with written informed consent from all subjects. The protocol was approved by the Institutional Review Boards of Vanderbilt University Medical Center and Kings College hospital.

## Author Contributions

VS collected the data, analyzed the data, prepared the manuscript, and designed the study. VK collected the data, analyzed the data, and designed the study. HdL, DM, VP, BS, WC, BE, StG, JG, AdK, SY, ShG, AuK, JM, GM, and AP treated the patients and reviewed the manuscript. MK kept follow up of patients. MJ designed the study, reviewed the data, and prepared the manuscript. KR designed the study, reviewed the data, and prepared the manuscript.

### Conflict of Interest Statement

The authors declare that the research was conducted in the absence of any commercial or financial relationships that could be construed as a potential conflict of interest.
