# Supplementary material for: Differential Interaction of Peripheral Blood Lymphocyte Counts (ALC) With Different in vivo Depletion Strategies in Predicting Outcomes of Allogeneic Transplant: An International 2 Center Experience
Source: Front Oncol. 2019 Jul 10;9:623. doi: 10.3389/fonc.2019.00623 (PMC6636242; doi:10.3389/fonc.2019.00623)
Supplement: Supplementary file 1 [file Table_1.docx]

|  | **NRM** | **Acute GVHD (all grades)** | **cGVHD (mild-moderate)** | **Severe cGVHD** | **CMV reactivation** | **EBV reactivation** |
| --- | --- | --- | --- | --- | --- | --- |
| **ALC (day 2)** | 0.11 | 0.6 | 0.84 | 0.60 | 0.86 | 0.55 |
| **ALC (day 2) interaction with alemtuzumab dose** | 0.14 | 0.6 | 0.83 | 0.66 | 0.83 | 0.53 |
| **Supplementary table 1**: Univariate analysis for ALC (day 2) and ALC (day 2) interaction with alemtuzumab dose in predicting outcome  NRM- Non-relapse mortality, GVHD- graft vs host disease, CMV- Cytomegalovirus, EBV- Ebstein Barr virus. | | | | | | |

Supplementary Figure 1 and 2

Figure 1-

A and B- The 5-year OS and DFS (protocol 1- ATG, protocol 2-alemtuzumab).

C-Cumulative incidence of any grade chronic GVHD.

D- The cumulative incidence chronic severe GVHD.

Figure 2-

A- Cumulative incidence of non-relapse treatment-related mortality (NRM).

B- Cumulative incidence of relapse (protocol 1 –ATG, protocol-2, alemtuzumab). Relapses were

significantly lesser in group 2, (p=0.04, HR-1.4, CI, 1.0-2.0),
