# Supplementary figures and images for: Differential Interaction of Peripheral Blood Lymphocyte Counts (ALC) With Different in vivo Depletion Strategies in Predicting Outcomes of Allogeneic Transplant: An International 2 Center Experience
Source: Front Oncol. 2019 Jul 10;9:623. doi: 10.3389/fonc.2019.00623 (PMC6636242; doi:10.3389/fonc.2019.00623)

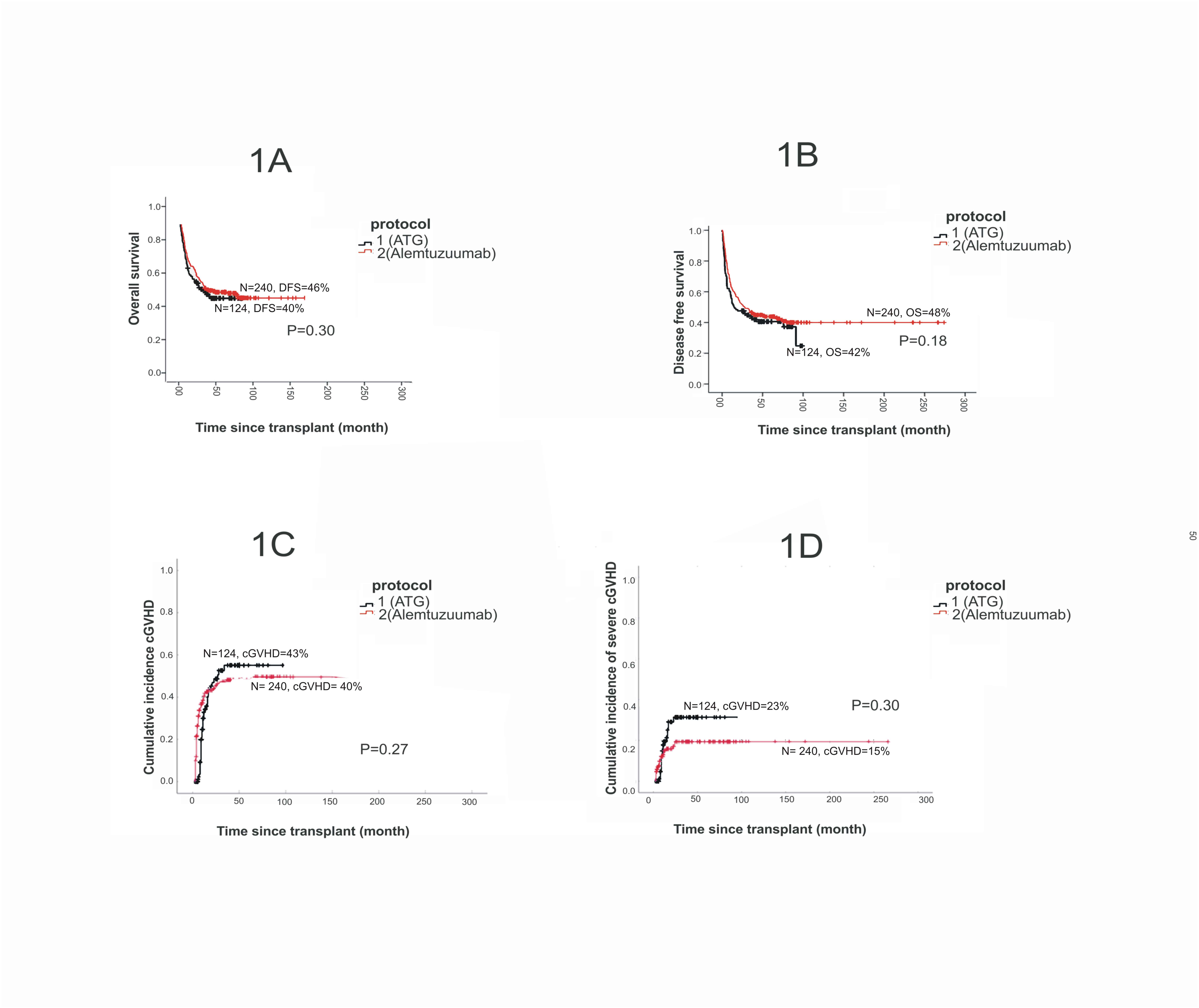

Supplement: Supplementary file 2 [file Image_1.JPEG]
